# Are medications with anti-cholinergic properties prescribed and reviewed appropriately on a male older person's organic ward?

**DOI:** 10.1192/bjo.2021.250

**Published:** 2021-06-18

**Authors:** Ben Greenhalgh, Hina Anwar, Rosemary Hedley, Celine Perkins, Rachel Shaw

**Affiliations:** Monkwearmouth Hospital, Sunderland, Cumbria, Northumberland Tyne Wear NHS Foundation Trust

## Abstract

**Aims:**

Patients admitted to Roker ward (male organic psychiatric ward) should have a decreased anticholinergic burden of medication on discharge compared to admission. This will be demonstrated by a reduced score on the Anticholinergic Cognitive Burden (ACB) scale on discharge compared to admission. Target: 80%.

Where new medicines with anticholinergic burden are prescribed during admission, there should be evidence that the anticholinergic properties of these medications have been considered prior to prescribing (via documentation in care co-ordination reviews or progress notes). Target: 100%

**Method:**

Electronic records were searched for all discharges from Roker ward between 1/1/2019 – 31/12/2019. For each record the follwing information was recorded: demographics; primary diagnosis; total ACB score on admission; and total ACB score on discharge. For all new medications started with an ACB score of over zero, records were searched to establish whether there was evidence that the anticholinergic properties of these medications had been considered.

**Result:**

47 patients were identified who were discharged over the time period in question. 30 patients had no difference in ACB score between admission and discharge; 10 patients had a reduction in ACB score and 5 patients had an increase. A total of 9 new medications with ACB scores over zero had been started during all admissions; there were no occasions where there was documented evidence to show that the anticholinergic burden of these medications had been considered.

**Conclusion:**

27% of patients had a reduction in their total ACB score during admission; the target was 80%.

The reasons for starting medications with an ACB score of greater than 1 were documented in 0% of cases; the target was 100%.

As both targets were missed by a significant margin, it was recognised that there were significant areas for improvement. The following plan was therefore implemented:

1. Following discussion with the ward consultant and ward pharmacist, regular prescriber meetings have been set up which involve senior nursing staff, medical staff and pharmacy – anticholinergic burden is calculated for each patient as part of these meetings
2. A re-audit is recommended after 6 months.

